# Opinion: Yes

**DOI:** 10.1590/S1677-5538.IBJU.2015.05.02

**Published:** 2015

**Authors:** Stanley E. Althof

**Affiliations:** Executive Director, Center for Marital and Sexual Health of South Florida; Professor Emeritus, Case Western Reserve University School of Medicine

**Keywords:** Erectile Dysfunction, Therapeutics, PDE-5 Inhibitors

Prior to the US approval of sildenafil in 1998, the available treatment options for erectile dyfunction (ED) included: intraurethral alprostadil (MUSE), intracavernosal injections (tri mix, alprostadil), vacuum pump therapy, placement of a penile prosthesis, hormone replacement therapy and individual or couples psychotherapy (1–7). The approval of sildenafil, a phosphodiesterase type 5 inhibitor (PDE5i), dramatically changed the treatment and research landscape. By utilizing sildenafil millions of men with ED could reliably and safely restore their erectile function. In addition to sildenafil, three other PDE5i medications have been approved in the US for the treatment of ED; they are: tadalafil (daily and as needed), vardenafil and avanafil.

While the PDE5i medications are successful in restoring erectile function in the majority of men, they are not as effective in men whose cavernous nerve has been damaged from a radical prostatectomy or in men with diabetes mellitus. Additionally, some men may not respond to PDE5i's because their vascular disease is too severe, they take concomitant medications that interfere with ED restoration, or they harbor severe psychological and/or interpersonal issues that overwhelm the prosexual effect of the drug. For men who utilize nitrate medications, PDE5i's are contraindicated because of their synergistic hypotensive effects, therefore these men must find other treatment options. For all the above reasons clinicians would welcome new agents that could overcome the limitations of the current PDE5i drugs.

I am certain that we will see new and better options for men suffering from ED. The introduction of the PDE5i's drugs revolutionized the manner in which we currently treat ED patients and opened the pathway for further research into the biological underpinnings of ED. One important limitation of the PED5i's is that they only provide short-term solutions to the chronic vascular issues that cause the ED. Treatments that would cure or reverse the underlying precipitating and maintaining factors would move us beyond the current standard of care.

Stem cell therapy is an exciting new treatment option that in theory offers the potential to reverse the underlying causes of ED and reduce patient reliance on the tran sient effects of the PDE5i drugs. It is also targeted at men with cavernous nerve injury or diabetic men whose response to PDE5i drugs is suboptimal.

## REVIEW OF STEM CELL THERAPY FOR ED

Stem cell therapy was initially based on the theoretical rationale that stem cells can differentiate into a range of cell types such as endothelial, smooth muscle, Schwann cells, and neurons8. Stem cells were delivered via intracorporal penile injections to replenish the depleted endothelial cells and/or caveronous smooth muscle cells. A different theoretical understanding is that stem cell therapy results in the host's regeneration, as opposed to simply replenishment, of endothelial and cavernous smooth muscle cells and is able to restore the interactions between these structures (8).

The vast majority of published studies focus on animal models with only one study in humans and one ongoing clinical trial in humans. Lin reports that intracavernous injected stem cells can escape the penis and hone into the bone marrow possibly accounting for systemic antidiabetic effects and prolonged restoration of erectile function (8).

## CONCLUSION

New and promising therapies for ED continue to evolve. The PDE5i's significantly advanced our understanding and ability to treat men suffering from ED. However, stem cell therapy may become the next generation of ED treatment offering the field of sexual medicine and our patients new possibilities. While it will take time to conduct the necessary human trials and obtain regulatory and ethical approvals, stem cell therapy may move us into the next wave of treatment options for ED.
